# Across species: A comparative perspective on red cell homeostasis and its influence on our understanding of human physiology and disease

**DOI:** 10.1111/bjh.70297

**Published:** 2026-01-04

**Authors:** Kathleen M. Connolly, Pengyi Ding, Rasiqh Wadud, David C. Rees, John N. Brewin, John S. Gibson

**Affiliations:** ^1^ Department of Veterinary Medicine Cambridge UK; ^2^ Department of Haematology King's College Hospital London UK

**Keywords:** cation homeostasis, comparative, macromolecular crowding, membrane transport, pathophysiology, pump‐leak, red cell, volume regulation

## Abstract

This review emphasises how studies on animal red cells have enriched our understanding of the behaviour of those from humans. For example, the pump–leak model for long‐term volume stability is indebted to work on high potassium (HK)‐ and low potassium (LK)‐containing sheep red cells. Studies in several species including trout have been useful for detailing how the co‐ordinated behaviour of red cell transport proteins is involved in shorter term volume homeostasis and other functions. Our understanding of how protein phosphorylation pathways control the activity of the cation‐chloride cotransporters has been given impetus by work in rabbit, sheep, trout and other species. Red cells from dogs and cats were historically important for developing theories on macromolecular crowding and cation permeability. Cattle red cells have helped substantiate that band 3 is not essential for red cell integrity. Work in many other species has informed our understanding of red cell physiology and a discussion of these areas illustrates how a comparative perspective has resoundingly enhanced and enriched our knowledge of human red cell physiology. A similar comparative approach to red cell pathophysiology is much less comprehensive although it has the potential to be invaluable for a better understanding of problems in humans.

## Graphical abstract

This review highlights how studies on animal red cells have enriched our understanding of the behaviour of those from humans. A comparative perspective has enhanced our knowledge of human red cell pathophysiology

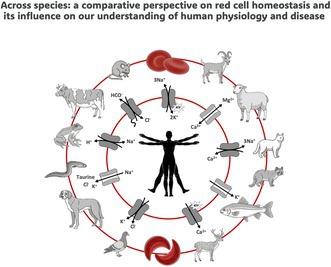

## INTRODUCTION

Free haemoglobin is toxic, reacting with essential regulatory molecules like nitric oxide to cause widespread vascular dysfunction and severe renal damage. A primary role of red cells in all vertebrate species is to safely contain haemoglobin in a protected environment, as well as to facilitate the transport of blood gases.^1^ Not surprisingly, red cells from different species share many features and challenges in common (Table 1). However, there are also some striking differences.

**TABLE 1:** Red cell physiological challenges.

Challenges	Causes	Evolutionary adaptations
Long‐term volume regulation	Osmotic imbalance from impermeant intracellular solutes, often negatively charged	Pump–leak model; often using the Na^+^/K^+^ pump; Ca^2+^ pump plus Na^+^/Ca^2+^ exchange in carnivores
Short‐term volume regulation	Anisotonicity in epithelia blood vessels like the renal medulla; isotonic load (β‐NHE of trout); euryhaline vertebrates	Regulatory volume transporters and channels for regulatory volume increase and decrease (RVI/RVD). Not humans
Energy supply	ATP for pumps (Na^+^/K^+^ pump, Ca^2+^ pump); GSH synthesis; protein phosphorylations; hexokinase	Glycolysis using glucose in most mammals; inosine in some (pigs); mitochondria in lower vertebrates
Oxidative threat	Intracellular from haem Fe^2+^ / methaemoglobin; reactive oxygen species; extracellular from activated white cells and endothelia	Cytoplasmic GSH, SOD, catalase; membrane peroxiredoxin; vitamins C and E
Shape, malleability and strength for reversible shape changes	Circulatory shear stress; narrow blood vessels; splenic slits	Cytoskeleton of spectrin tetramers & actin bound to the plasma membrane via band 3 and glycophorin. Not agnathans
Oxygen transport & delivery	Respiration; pulmonary; transplacental; across fish gills; environmental hypoxia	Increase fetal oxygen affinity (except cat)—Hb isoform & Bohr effect (many); low 2,3‐BPG affinity (e.g. primates), low 2,3‐BPG levels (horse, pig, dog), low 2,3‐BPG levels & affinity (ruminants, cats). Fall in red cell pH postnatally (sheep, goats). Adrenergic increase red cell pH (β‐NHE in teleosts). ATP (fish, reptiles), ATP / 2,3‐BPG (amphibia), ITP (birds) in lower vertebrates
Carbon dioxide transport & delivery	Respiration; intracellular bicarbonate production; carbonic anhydrase; Hb buffering	Band 3—except Japanese cattle and agnathans—for access to plasma water

*Note*: The table summarises some of the commoner physiological challenges faced by red cells, together with some of the evolutionary solutions. Transplacental oxygen delivery in mammals and regulatory effect of organic phosphates are particularly interesting examples of convergent evolutionary solutions.^2^ GSH, reduced glutathione; SOD, superoxide dismutase. Other details and references can be found in the text.

Gulliver illustrated the diverse morphology of red cells across the animal kingdom in 1840.^3^ The largest is probably that of the aquatic salamander *Amphiuma*, with a remarkable diameter of about 0.1 mm, visible to the naked eye. Red cells from humans are about one‐tenth of this size, 8 μm; those of the mouse deer are probably the smallest, 1.5 μm.^4^ Other structural differences are evident, most obviously that mature red cells from lower vertebrates retain their nucleus, while those of mammals do not. Functional properties, too, are diverse (Table 1).

Despite such differences—or may be because of them—work on animal red cells has enriched our understanding of human red cell function. The value of a comparative approach in haematology is evident from consideration of a few historical landmarks. Gulliver was also the first to observe red cell sickling in deer; some 70 years before, Herrick saw sickle cells in human patients.^5^ Arthropod vectors for erythrocytic parasites were first seen in about 1890, in cattle shown to contract Texas red water fever, a babesiosis, from ticks^6^; several decades before mosquitoes were shown to carry malaria. The seminal pump–leak model of cellular volume control was first established in sheep red cells.^7^

This article emphasises how work from other species has contributed to our understanding of homeostasis—mainly cation balance—of human red cells.^1^ Some of the challenges to which red cells are exposed are summarised in Table 1.

## SHEEP

All cells are subject to the constant threat of osmotic swelling from the intracellular presence of impermeable solutes. These are often negatively charged and include haemoglobin (Hb) (*c*. 5 mM or 350 g.L^−1^) and high concentrations of smaller metabolites (*c*.100 mM) including organic phosphates like adenosine triphosphate (ATP) and 2,3‐biphosphoglycerate (2,3‐BPG) and organic osmolytes. In the absence of a protective strategy, these impermeable solutes would cause unbalanced osmotic water influx with eventual haemolysis. Nevertheless, most cells, including red cells, present with a stable characteristic volume. Human red cells have a volume of about 90 fl, a surface area of 140 μm^2^ and a mean cell haemoglobin concentration (MCHC) of about 320–360 g.L^−1^ ^8^ Their volume is about 0.55–0.60 of the maximal spherical volume, allowing them to assume their characteristic biconcave shape and an optimal surface area‐to‐volume ratio enabling them to distort and squeeze through narrow blood vessels. The rheology of larger or smaller red cells becomes compromised by steric hindrance or through increased viscosity.

Bacteria and plant cells have a rigid cell wall or cytoskeleton to offset osmotic water gain and use an elevated intracellular hydrostatic pressure—sometimes to markedly high values—to oppose swelling.^2^, ^3^, ^4^, ^7^, ^8^, ^9^ Animal cells lack this option as their plasma membrane is thin and flexible and unable to withstand significant pressure gradients. Instead, they must have recourse to energy‐requiring osmolyte efflux to counteract osmotic water influx—the ‘pump–leak’ model (PLM). The first full articulation of this model to red cells is credited to Tosteson and Hoffman^7^ building on the earlier foundations of van't Hoff, Donnan, Krogh, Davson, Dean, Conway and others. At around the same time, came the realisation that red cell membranes contain an enzyme with an Mg^2+^‐ATPase activity stimulated by Na^+^ and K^+^ ions. This is the ATP‐driven Na^+^/K^+^ pump, the pre‐eminent ‘pump’ of the pump–leak model, found in most animal cells.

In the 1950s, both Tosteson and Hoffman were working in Cambridge, UK—Tosteson with Hodgkin in the Physiological Laboratory, measuring the membrane potential of red cells; Hoffman nearby, in Roughton's Department of Colloid Science, while also collaborating with Whittam on red cell ghosts in the same Department as Tosteson. A particularly significant impetus, however, occurred back in the United States, in Robert Berliner's laboratory at the Bethesda National Institutes of Health, where they were encouraged to work on sheep red cells.^10^ Sheep and some other ruminants have individuals genetically dimorphic for red cell cations. Red cells from high potassium‐containing (HK) sheep are rather like those from humans with an intracellular K^+^ of *c*. 90 mM and Na^+^ of *c*. 10 mM; those from low potassium‐containing (LK) ones have opposite cation gradients, with an intracellular K^+^ of about 10 mM and Na^+^ of 90. Both solve the problem of osmotic water influx using an ATP‐driven pump to mediate Na^+^ efflux and K^+^ influx, but with different kinetic solutions.

**FIGURE 1:**
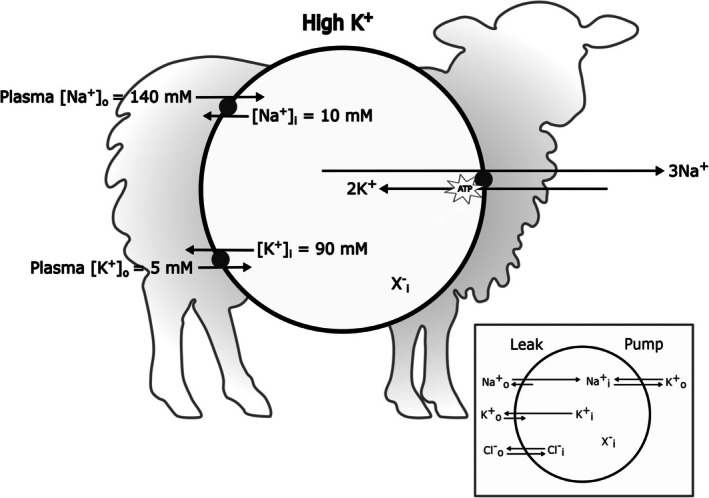
Maintenance of red cell volume by the pump–leak model in sheep red cells. Figure 1 is taken from Tosteson & Hoffman's paper on ‘regulation of cell volume’.^7^ They used a set of theoretical equations to solve for [Na^+^], [K^+^] and [Cl^−^] in high K^+^‐containing (HK) and low K^+^‐containing (LK) sheep red cells to show how maintenance of red cell volume could be achieved by an ‘exchange pump in parallel with diffusive leaks’.^7^ They then empirically measured both the ‘pump’—as a strophanthidin‐sensitive flux—and the ‘leak’ fluxes with ^42^K^+^ and ^24^Na^+^. Both types of sheep conformed to their theoretical ‘pump–leak model’. However, pump fluxes are about fourfold higher in HK sheep red cells and the ratio of the pump/leak rate constants is about 10‐fold higher: Pump/leak ratios (β) are about 15 in HK sheep versus 1.5 in LK sheep, and Na^+^/K^+^ leaks (α) are 0.5 in HK sheep and 0.2 in LK sheep, balancing the osmotic effect of impermeable intracellular solutes (X^−^). With the prevailing plasma levels of Na^+^ and K^+^ of 140 and 5 mM in both sheep genotypes,^11^ these values result in intracellular cation values, respectively, of about 90 mM K^+^ and 10 mM Na^+^ in HK sheep red cells, and the reverse in LK sheep. Later, the stoichiometry of the pump was established as 3 Na^+^: 2 K^+^: 1 ATP (as shown). The ‘leaks’ have since been identified as specific transport pathways—that for K^+^ in LK sheep red cells is mainly manifested by the swelling‐activated KCl cotransporter (KCC).^12^, ^13^, ^14^ The main diagram shows cation balance for a red cell from an HK sheep in which the higher pumping capacity coupled with a modest leak results in high intracellular K^+^ and low intracellular Na^+^. A lower pumping capacity and relatively larger leak reverse the cation contents in red cells from LK sheep. The inset is a re‐drawing of Tosteon & Hoffman's original figure. In sheep, the HK/LK cation polymorphism appears to be determined by single gene encoding the M and L red cell antigens. These antigens, and antibodies to them, appear to regulate the activities of both the Na^+^/K^+^ pump and the KCC, but the mechanism remains unsolved—despite the pivotal role of these animals in establishment of the seminal pump–leak hypothesis. KCC and other cation‐chloride cotransporters (CCCs) are controlled by protein phosphorylation and O_2_ tension is a particularly important regulatory modality—see text for details.

Tosteson and Hoffman modelled the situation using an a priori mathematical analysis, echoing the Hodgkin–Huxley model on nerve action potentials. A handful of elegant differential equations formulated from physico‐chemical first principles provided solutions for membrane potential, osmosis, flux of the permeable ions (Na^+^, K^+^, Cl^−^) and cell volume. They then empirically measured the pump and leak characteristics of red cells from sheep of both genotypes using radioactive tracers. Their experimental data fitted the theoretical model of how the solution to osmotic balance was achieved. Both LK and HK sheep red cells use the pump–leak model for cation and volume homeostasis: HK sheep red cells have a high Na^+^/K^+^ pump activity and high passive Na^+^ leak; LK sheep, a low pump rate, with a smaller Na^+^ leak but a larger K^+^ leak^7^ (Figure 1). Later, this pump–leak model was confirmed for human red cells^15^ and other cell types.

Active ATP‐driven removal of solutes with a pump is a necessity for osmotic balance in animal cells. While, in red cells from many species, the pre‐eminent Na^+^/K^+^ pump balances the osmotic attraction due to the impermeable species, the pump does not have to involve either of these ions. Mature dog and cat red cells, for example, notably lack an Na^+^/K^+^ pump and rather use an ATP‐driven Ca^2+^ pump,^16^ as discussed later.

Variants of the pump–leak mechanism provide long‐term volume stability but cannot ensure a return to the original volume after perturbation.^10^ Notwithstanding, red cells from many species—although not mature human ones—are able to restore their volume in the short term. They have mechanisms to shrink following swelling and to regain volume after shrinkage—termed regulatory volume decrease (RVD) or increase (RVI)—as discussed in subsequent sections.

## TROUT

Red cells from lower vertebrates and non‐human mammals have been particularly important in developing our understanding of how integration of transporter function is critical for homeostasis. Trout red cells, for example, have long been used as a model for short‐term volume regulation. The historical foundations underpinning much of this work were initiated at the Jean Maetz Laboratoire, Villefranche‐sur‐Mer, where there was a focus on ion and water homeostasis in fish. Maetz's work was continued by Motais, Borgese, Guizouarn and others,^17^, ^18^, ^19^ and then later at the Station Biologique de Roscoff co‐ordinated by Thomas, Egée, Bouyer and colleagues.^20^, ^21^, ^22^, ^23^, ^24^, ^25^

Red cells from trout show a powerful Na^+^/H^+^ exchanger responsive to β‐adrenergic stimulation, the β‐NHE (Na^+^/H^+^ exchanger),^26^, ^27^, ^28^ which uses the inward Na^+^ gradient to remove intracellular H^+^ ions. The subsequent intracellular alkalinisation increases both the O_2_ affinity of Hb—similar to the more familiar Bohr effect—and also the total O_2_‐binding capacity. Acidification does the opposite—the Root effect^1^—which is used physiologically to deliver O_2_ to the swim bladder and rete retinalis/choroid rete mirabile.^29^, ^30^

**FIGURE 2:**
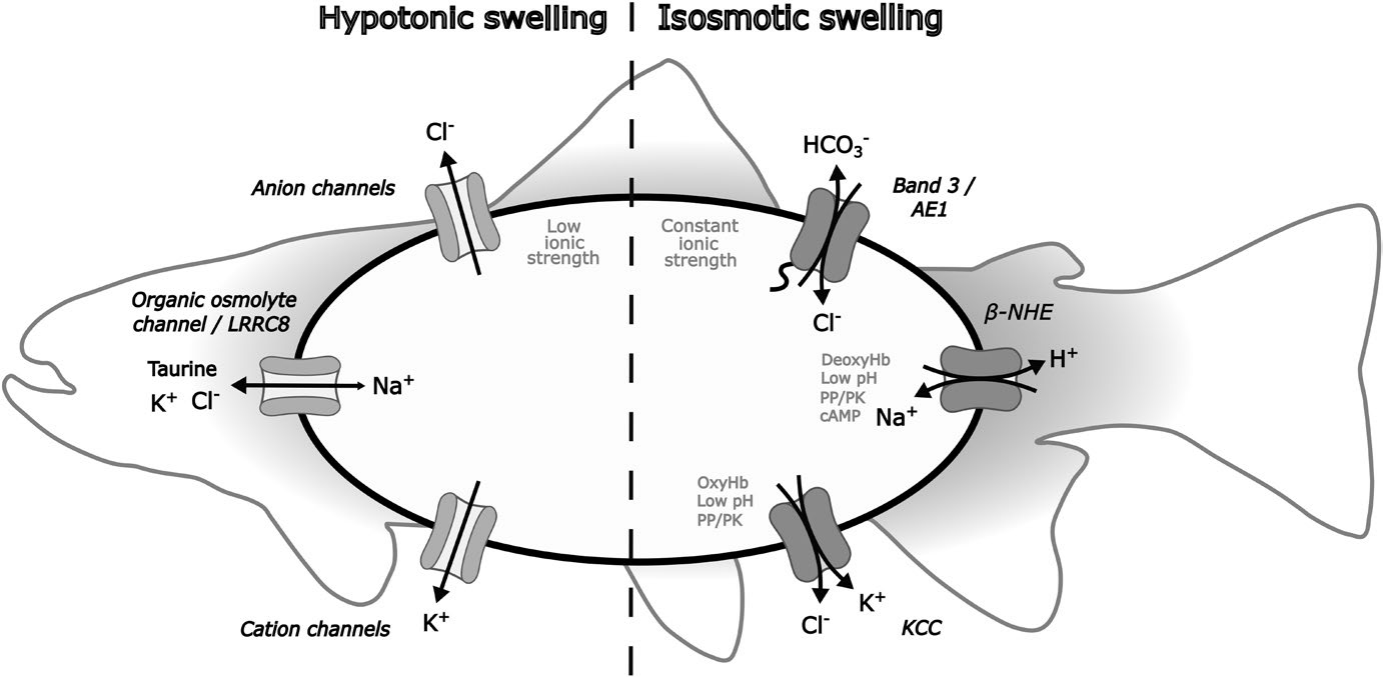
The mechanism of volume regulation in trout red cells following swelling. Trout red cells, which are more ellipsoidal than circular in profile, have a powerful Na^+^/H^+^ exchanger activated by β‐adrenergic stimuli via elevation of intracellular cAMP (β‐NHE).^26^, ^27^, ^28^ When coupled with Cl^−^ entry in exchange for HCO_3_^−^ on band 3, the result is a net gain of NaCl and isosmotic swelling. Under these conditions, volume recovery (regulatory volume decrease, RVD) is mainly through activation of the KCl cotransporter (KCC).^32^ By contrast, when swollen hypotonically, RVD is mainly via solute loss through channel(s) mediating the efflux of K^+^, Cl^−^ and organic osmolytes such as taurine and sorbitol, and influx of Na^+^.^17^, ^18^, ^19^, ^32^, ^33^, ^34^ A volume‐sensitive organic anion channel (VSOAC), also called a volume‐regulated anion channel (VRAC) or a volume‐sensitive outwardly rectifying anion channel (VSOR), which is also observed in malaria‐infected red cells, participates.^22^, ^35^, ^36^ Under low ionic strength conditions, the volume‐sensitive channels participating in solute loss are often members of the LRRC8 family, which are probably the molecular identity of the proteins mediating VSOAC/VDAC/VSOR activity in many different cell types.^37^, ^38^ O_2_ tension is also important in regulating red cell ion permeability, as first seen in avian red cells.^39^, ^40^ Trout red cells proved useful in identifying that oxy‐deoxy transitions of haemoglobin co‐ordinately stimulated NHE and inhibited KCC^41^, ^42^ (deoxyHb/oxyHb). In trout, the stress of hypoxia leads to adrenaline release, stimulating red cell β‐Na^+^/H^+^ exchanger through cAMP and deoxyHb. Elevation of intracellular pH both increases the O_2_ affinity and O_2_‐binding capacity of Hb to offset the reduction in O_2_ carriage which would otherwise result. In the rete of swim bladders and the retina, acid secretion reverses the effect to secrete O_2_ into these tissues—the ‘Root effect’.^1^ Cation chloride cotransporters (CCCs), in particular, are often regulated by protein kinases (PKs) and their conjugate protein phosphatases (PPs)—see text for details.

In conjunction with Cl^−^ uptake via band 3, activation of the β‐NHE also leads to gain of NaCl and isosmotic swelling with no change in intracellular ionic strength. In addition, and notwithstanding the osmoregulatory prowess of euryhaline teleosts, exposure to anisotonic environments can alter plasma osmolality.^31^ This is particularly relevant to migratory fish, like trout, eels and flounders which can move from hypotonic fresh rivers to hypertonic seawater. While plasma hypotonicity also causes red cell swelling, here there is a concomitant reduction in intracellular ionic strength. In both cases, trout red cells activate transporters to counter swelling (Figure 2).

In isosmotic swelling, solutes are mainly lost through coupled K^+^ and Cl^−^ efflux via the K^+^‐Cl^−^ cotransporter (KCC),^17^, ^18^, ^19^ a system used by red cells from many species (see Table 2). Swelling‐stimulated KCC was actually first appreciated in LK sheep red cells^12^, ^13^ and subsequently in humans,^46^, ^47^ oddly, perhaps, as in neither does it result in volume‐regulatory solute losses. In LK sheep, there is little chemical gradient to drive net KCl movement; in humans, mature red cells have a quiescent KCC^47^ although it can be activated pharmacologically, for example, by *N*‐ethylmaleimide.^46^ How KCC activity is coupled to change in red cell volume is considered later.

**TABLE 2:** Physiology and pathology of red cell potassium chloride cotransporters.

Property	Features
Presence in red cells	First recognised as a Cl^−^‐dependent/‐activated cation flux, later as a cotransporter
Volume sensitivity	Swelling‐activated, shrinkage‐inhibited; sometimes volume regulatory, less so in humans; volume reduction of erythroblasts
Red cell age	Young, less dense human red cells only; quiescent when mature though activated pharmacologically
Thiol oxidation	*N*‐ethylmaleimide activates, even in mature red cells when physiologically quiescent
pH dependence	Bell‐shaped response; maximal at c. pH 7; intracellular pH cf. extracellular
Protein phosphorylation	Okadaic acid & calyculin A inhibit; staurosporine activates; genistein activates
Sickle cell disease	High and abnormal activity contributes to shrinkage & polymerisation; HbSS and especially HbSC
Other haemoglobin variants	Very high levels, altered charge on Hb
Magnesium dependence	High inhibits; low stimulates
Urea stimulation	Levels in renal medulla
Oxygen sensitivity	Arterial PO_2_s activate, hypoxia inactivates, nadir at O_2_ P_50_ for HbSS; differences between HbAA, HbSS and HbSC
Cloning in red cells	Through homology with other cation‐coupled Cl^−^ cotransporters
Phosphoresidues	Inhibition by phosphorylation (residues T991/1048)
Inhibition by WNK in epithelia	WNKs inhibit via SPAK/ORS1
Role of WNK in red cells	DeoxyHb stimulates NKCC via WNK1; ?inhibits KCC; pan‐WNK inhibitors stimulate KCC

*Note*: The family of chloride cation cotransporters has many important roles in red cell pathophysiology, as exemplified here by the KCl cotransporter (KCC). Some important features of KCC are summarised. Further details and references can be found in the text and more comprehensive reviews.^43^, ^44^, ^45^

By contrast, RVD following hypotonic swelling of trout red cells uses mainly increased activity of channels for K^+^, Cl^−^ and organic osmolytes like taurine.^17^, ^19^ A role for band 3 was proposed^17^, ^19^—either acting as a non‐selective channel rather than in its more established transport role as an anion exchanger or perhaps acting to regulate the activity of other membrane proteins. Gradually, the existence of volume‐activated ‘organic anion channels’ became established (Figure 2). Their molecular identity is likely that of the LRRC8 channel^37^ whose activity is increased in low ionic strength media,^38^ while that of KCC is decreased, as in trout red cells.^19^ Ionic strength or some function of it, such as electrostatic forces or Cl^−^ concentration, represents an important modality in volume responses, along with membrane distortion and macromolecular attractions—see later.

The Roscoff group have also been interested in direct mechanical deformation of the red cell, using electrophysiological techniques to study single cells.^20^ Their work has extended to malaria‐infected red cells, initially from birds^21^ and then also from humans.^22^, ^23^, ^24^ Deformation of the red cell activated several different cation and anion channels.^25^, ^33^, ^34^ In humans, channel activity was dependent on extracellular Ca^2+^ ^25^ Their results were consistent with transient Ca^2+^ entry via a mechanosensitive pathway leading to activation of K^+^ efflux via the Ca^2+^‐activated K^+^ channel or Gárdos channel.^48^ Normally, this channel is quiescent because the high capacity plasma membrane Ca^2+^ pump (or PMCA) keeps intracellular Ca^2+^ levels below that required for activity. Dyrda et al. postulated that Ca^2+^ entry following mechanical distortion was sufficient to transiently overwhelm the PMCA, thus allowing brief Gárdos channel activation. They further surmised that conductive K^+^ loss through this pathway overcomes the normal anion selectivity of the red cell membrane, hyperpolarising the membrane, with subsequent opening of separate anion channels.^25^ Subsequently, identification of the mechanosensitive channel, PIEZO1, in red cells has provided a molecular basis for Ca^2+^ entry,^49^ reconciling with earlier reports of Ca^2+^ entry following shear stress.^50^, ^51^

Mature human red cells lack the capacity for protein synthesis. Accordingly, as they age, the activities of the Na^+^/K^+^ pump and PMCA decline, as does glycolytic ATP synthesis. Na^+^ gain and dissipation of Na^+^ and K^+^ gradients would jeopardise pump–leak volume stability. It may be thought that these age‐dependent perturbations of the pump–leak model would result in cell swelling, as Na^+^ efflux via the Na^+^/K^+^ pump fails to keep up with entry. In fact, human red cells usually show a modest dehydration with age, until a terminal reversal and removal. Understanding the integrated behaviour of these numerous permeability pathways is complicated but has benefited from the computer simulations of red cell behaviour developed by Lew and colleagues.^52^ Their red cell model comprises a more complete set of ion transporters than those of the simple pump–leak model of Tosteson and Hoffman, notably band 3 (see later). It includes other constraints such as the non‐ideal osmotic behaviour of haemoglobin and the H^+^‐binding properties of impermeant solutes and has also been updated to incorporate the findings from Roscoff.^25^, ^53^, ^54^, ^55^, ^56^

The model suggests that, upon deformation, such as experienced in normal red cells squeezing through narrow blood vessels, Ca^2+^ entry occurs via PIEZO1 with subsequent activation of Gárdos and anion channels, as observed by Dyrda et al. This would result in transient bursts first of CaCl_2_ gain followed by KCl loss, resulting in tiny (‘quantal’ or ‘infinitestimal’) intermittent net shrinkage.^54^ While it is tempting to hypothesise that repeated losses of small pulses of KCl as red cells squeeze through narrow capillaries at each circulatory transit would accumulate and account for their progressive modest dehydration with age, the model in fact predicts that this ceases after a few days.^53^, ^55^ Both quantal changes and senescent decrease in Na^+^/K^+^ pump and PMCA activities are required for the observed progressive reduction in red cell volume, and its late reversal just prior to red cell removal.^55^

Lew's model explains how the co‐ordinated activity of many membrane transport proteins—PIEZO1, PMCA and the Gárdos channel and also others like K^+^‐Cl^−^ cotransport, Na^+^/K^+^ pump and band 3—interacts, sometimes unpredictably, to determine normal red cell behaviour, including their longevity.^55^, ^56^ Recently, experimental evidence has been obtained which supports some of these mechanisms.^57^ Abnormal activities of any of these major participants would interfere with normal volume and hence lifespan, as in some human haemolytic anaemias^58^, ^59^—as discussed briefly in the last section. The model also raises a caveat for the relevance of transgenic mice of red cell disorders, as the behaviour of the murine red cell transporters, cation contents, cell volumes and details of the vasculature may differ from the human situation.

## DOG

**FIGURE 3:**
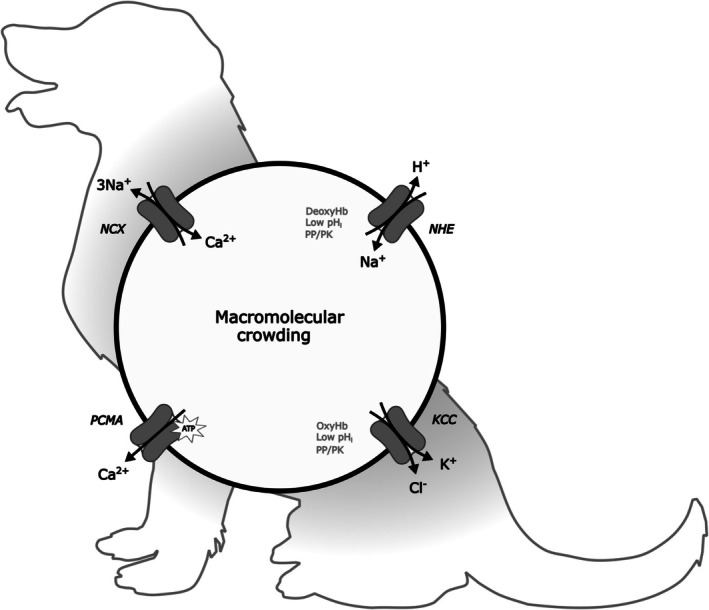
Volume regulation of dog red cells and macromolecular crowding. Mature dog red cells, like those from low K^+^‐containing (LK) sheep, lack an Na^+^/K^+^ pump and intracellular cations are low in K^+^ and high in Na^+^. They solve the problem of osmotic swelling due to the presence of impermeant intracellular solutes by coupling Ca^2+^ influx via the Na^+^/Ca^2+^ exchanger (NCX or NCE) with an ATP‐driven Ca^2+^ pump (PMCA).^16^ Like trout red cells, they can also respond to volume perturbation: swelling stimulates NCX and also the KCl cotransporter (KCC), although the latter cannot mediate substantial solute loss in LK cells; shrinkage stimulates the Na^+^/H^+^ exchanger (NHE).^61^, ^62^ The volume sensor has been much studied using dog red cell ghosts in which haemoglobin concentration and volume can be independently varied.^63^, ^64^, ^65^, ^66^ These studies led to the hypothesis that the ‘volume sensor’ was due to alterations in protein activity through the proximity of other macromolecules, ‘macromolecular crowding’.^67^ Recent work in kidney epithelia has shown that shrinkage activates a WNK1 kinase which inhibits KCC but stimulates NKCC^68^, ^69^ via a mechanism involving non‐membrane biomolecular condensates created through phase separation. Less is known about WNK1 in red cells although it does appear to inhibit KCC following volume reduction in human red cells.^70^ More recently, WNK1 has been shown to participate in O_2_ sensitivity of red cell NKCC,^71^ stimulating the transporter when it is displaced from band 3 by deoxyHb. It is likely to be involved in the opposite response of KCC to oxy‐deoxy Hb transitions (but cf.^72^).

John Parker observed in the 1970s that the ‘early history of cation permeability of red cells is written in the blood of dogs and cats’.^16^ In these species, and ruminants, bears and a few others,^60^ fetal and immature red cells resemble those of humans with high intracellular K^+^ and low Na^+^ levels maintained by an Na^+^/K^+^ pump. In older red cells, the Na^+^/K^+^ pump disappears and cells assume low K^+^ and high Na^+^ contents—like LK sheep. In dogs and cats, the pump–leak mechanism remains fundamental but is solved differently: the efflux of Na^+^ occurs via a secondary active Na^+^/Ca^2+^ exchanger (NCX or NCE), coupled functionally to active Ca^2+^ removal by PMCA^16^ (Figure 3).

Dog and cat red cells, like those of trout, also respond to volume change. Swelling stimulates efflux of Na^+^ via NCX and K^+^ via KCC, while shrinkage increases Na^+^ influx through NHE^61^, ^73^ (Figure 3). These opposing volume regulatory systems, as in red cells from many other species, show co‐ordinate regulation by a number of stimuli,^74^ notably protein phosphorylation.

Red cells from most species express KCC, one of a family of electroneutral cation‐coupled chloride cotransporters (CCCs)—see Table 2. Many were cloned in the 1990s,^43^ first in the urinary bladder of the winter flounder which is a rich source of the salt absorbing, thiazide‐sensitive, Na^+^‐Cl^−^‐coupled cotransporter (NCC). Subsequently, others were cloned by homology to NCC in other tissues and other species, notably from the mammalian kidney. In human red cells, the CCCs family includes the Na^+^‐K^+^‐2Cl^−^ cotransporter (NKCC) as well as KCC.^75^ As in many tissues, Na^+^‐coupled cotransporters like NKCC mediate solute gain and swelling, that is regulatory volume increase (RVI); KCC, the opposite (RVD).

For many years, it has been known that KCC and NKCC are regulated by protein phosphorylation. For KCC, the final event upstream to transporter activity appears to be a serine–threonine dephosphorylation, but tyrosine phosphorylation is also involved.^76^, ^77^ Uncovering the details has involved work in red cells from many species (see Table 2). Kinetic arguments and the effect of protein phosphatase inhibitors like okadaic acid and calyculin A—first from the work of Jennings and others in rabbit red cells^78^, ^79^ and then extended to other species including humans—led to the proposal that swelling inhibits a protein kinase (‘v’—or volume—kinase). Dephosphorylation is carried out by a constitutively active conjugate protein phosphatase(s), likely PTP1B.

How a protein phosphorylation cascade responded to volume change was unresolved for many years. Dogs and cat red cells proved particularly useful here. The work of Parker, Colclasure, Minton and others^63^, ^64^ emphasised a possible role for the altered activity of proteins resulting from the proximity of other macromolecules. Evidence came from red cell ‘ghosts’ made by hypotonic lysis and then resealing, so that the cell contained different concentrations of Hb and whose volume could be altered independently.^65^, ^66^ These experiments identified protein concentration as central to the activities of major volume regulatory pathways, including KCC and NHE,^80^ a mechanism termed ‘macromolecular crowding’.^67^, ^81^ This mechanism adds to membrane distortion (e.g. PIEZO1) and ionic strength (e.g. LRRC8 and KCC) as an important determinant of activity of membrane transporters in response to altered volume.

Further advances on how macromolecular crowding may impact the activity of CCCs like KCC have depended on work in epithelial cells such as kidney. Here, too, control involves protein phosphorylation, notably by with no‐lysine‐kinase (WNK1) ,^82^ which inhibits renal KCC while stimulating NKCC. Shrinkage acts on the WNK1 signalling pathway via macromolecular crowding, involving ‘biomolecular condensates’.^68^, ^69^ These are membraneless microdomains formed via phase separation—as when a poorly mixed salad vinaigrette disagreeably separates into oil and vinegar phases, though the properties of mixtures of cytoplasmic proteins are rather more complex.

Hyperosmotic shrinkage, or elevation of the intracellular concentration using eg Ficoll, encourage WNK1 to transition from a diffuse cytoplasmic distribution to a punctate arrangement. The result is increased WNK1 activity and phosphorylation‐induced inhibition of KCC and stimulation of NKCC, sometimes involving downstream SPAK and ORS1. The large C‐terminal domain of WNK1 has an intrinsically disordered region of low amino acid diversity. This site is conserved across species and is critical for crowding‐induced phase transition.^68^ The WNK pathways in red cells are less well investigated, but certainly WNK1 can inhibit KCC3 during volume reduction.^70^ In addition, pan‐WNK inhibitors are among the most powerful stimuli for KCC activity in normal and sickle human red cells.^83^ Hence, the pathways are demonstrably present.

Red cell O_2_ tension is also a powerful regulator of CCC activity.^39^ Deoxygenation inhibits KCC—as observed in early work in trout red cells^41^, ^42^—while stimulating NKCC and NHE. The molecular switch for NKCC is preferential binding of deoxyHb to the terminus of band 3, thus displacing WNK1 and stimulating NKCC activity.^71^ In a similar way, increased WNK activity would be expected to inhibit KCC in deoxygenated red cells^39^, ^42^ though this remains to be unambiguously proven. How reversible O_2_ binding to Hb affects the volume occupied by this protein and so altering macromolecular crowding is also relevant here but has not been investigated, and may differ between species, for example, crucian carp.^72^

In dog red cells, high ionic strength moves the volume set point of transporters like KCC towards lower volumes.^84^ High ionic strength also inhibits WNK1 and thereby removes inhibition of KCC, allowing activity to occur at lower volumes. Volume‐sensitive LRRC8 channels behave in a reciprocal manner such that, at high ionic strength, a larger cell volume is required for activity. These different behaviours could underlie the alternative mechanisms of RVD in trout red cells swollen either isosmotically or hypotonically.^17^, ^19^, ^37^

## CATTLE

Although absent from Tosteson & Hoffman's model, another key characteristic of the membrane of red cells from most species is band 3, the anion exchanger or AE1. In human red cells, it is present at a very high copy number of around 1.2 million.^8^ It is best known for mediating the exchange of HCO_3_^−^ for Cl^−^ and is very important—although not essential—for transport of CO_2_ in blood. In fact, the absence of band 3 in the agnathans^30^—and see below—provides an evolutionary adaptation to survival without this function.

In peripheral tissues, CO_2_ is hydrated inside the red cell to carbonic acid using carbonic anhydrase, after which the dissociated product, HCO_3_^−^, is exchanged on band 3 for extracellular Cl^−^—the Hamburger shift. The transporter gives lipid insoluble HCO_3_^−^ ready access to plasma water—the largest volume compartment of blood. As a consequence, most CO_2_ (*c*. 70%) in human blood is carried as HCO_3_^−^. In the lungs, the exchange mediated by band 3 reverses. HCO_3_^−^ re‐enters the red cell and Cl^−^ leaves, to generate gaseous CO_2_ for expiration. Band 3 therefore entrains intracellular pH of the red cell to that of plasma.^85^ These movements must also be completed within the time frame that red cells spend in the tissue and alveolar capillaries, rather less than a second—hence one of the reasons for the large copy number of band 3. In its functional absence—or when it is poisoned as, for example, in aspirin toxicity—more CO_2_ must be carried as the dissolved gas, necessitating a rise in the partial pressure of CO_2_, causing a respiratory acidosis and increased rates of alveolar ventilation.

**FIGURE 4:**
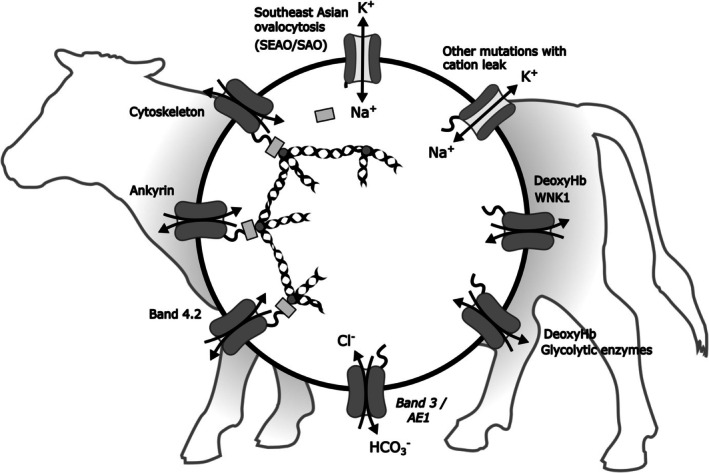
Multiple roles of band 3/AE1 in red cell pathophysiology. AE1 is present in a very large copy number, a consequence of which provides its alternative name, band 3—as the large third band in electrophoresis gels of red cell membranes. One of its major functions is anion exchange, of HCO_3_^−^ for Cl^−^, which is required to facilitate transport of CO_2_ between peripheral tissues and the lung. It also functions as an important component of the cytoskeleton.^86^ The binding, in particular, of ankyrin (light grey rectangle) and band 4.2 (dark grey circle) to its cytoplasmic tail—sometimes called cdb3^86^—connects the red cell membrane to the quasi‐hexagonal arrangement of spectrin tetramers (chains). Cytoskeletal integrity gives the red cell its strength and also its flexibility.^8^ This part of band 3 also binds other proteins including glycolytic enzymes and protein kinases and phosphatases including WNK1. Reversible preferential binding of deoxyHb over its oxygenated conformation can displace these proteins. Deoxygenation thereby increases glucose flux through the glycolytic pathway and reduces the activity of the pentose phosphate shunt, and hence synthesis of the antioxidant NADPH.^92^ It also weakens the cytoskeleton by displacing ankyrin which may be important in allowing red cells to squeeze through narrow capillaries in hypoxic tissues. DeoxyHb displacement of WNK1 stimulates the activity of the Na^+^‐K^+^‐2Cl^−^cotransporter (NKCC)^71^ and probably inhibits that of the K^+^‐Cl^−^ cotransporter (KCC). Mutations of band 3, such as the deletion in Southeast Asian Ovalocytosis (SEAO/SAO), can break important components of the cytoskeleton and thereby alter red cell shape and increase its fragility.^93^ Rare mutations, including that of SEAO/SAO, may also convert the anion exchange function of band 3 into cation leaks, for example, in some cases of hereditary stomatocytoses.^59^, ^94^, ^95^ Lack of band 3 in some Japanese black cattle and some human patients, nevertheless, is compatible with life.

The red cell also makes use of the very high copy number of band 3 for many other functions, notably for stabilising the cytoskeleton and for binding of other important proteins including glycolytic enzymes.^86^, ^87^, ^88^ It forms a key (‘vertical’) attachment point between the underlying (‘horizontal’) reticular network of largely spectrin tetramers and the enveloping plasma membrane.^86^, ^89^, ^90^, ^91^ Many other proteins participate, but the binding of ankyrin and band 4.2 to band 3 is critical (Figure 4). The majority of the cytoskeletal interactions are non‐covalent which stabilises the characteristic biconcave shape, while allowing them to distort reversibly as they squeeze through parts of the vasculature of smaller diameter. The plastic nature of the cytoskeletal interactions thereby allows the red cell to respond to the repeated shear stresses experienced during circulation, providing both flexibility and strength—some hundred times more flexible than latex of comparable thickness but structurally stronger than steel.^96^

The pivotal role of band 3 for cytoskeletal integrity is demonstrated by mutations in which it is unable to adequately fulfil this function. For example, 10%–20% of spherocytosis/elliptocytosis cases are due to band 3 mutants.^8^ One of the best known of these in humans is Southeast Asian ovalocytosis or SEAO/SAO.^93^ The mutation responsible for SEAO/SAO, like many red cell mutations, has accumulated because of a relative resistance to malaria. A deletion of the tail of band 3 causes misfolding and interferes with its association with ankyrin and other cytoskeletal proteins. Altered red cell morphology and fragility result, and heterozygous patients are markedly anaemic. In addition, the mutated band 3 may also function as a cation leak in this disease and in some hereditary stomatocytoses,^59^, ^94^, ^95^ which is reminiscent of the channel function responsible for RVD in trout red cells following hypotonic swelling^17^, ^19^ (see above and Figures 2 and 4).

Although SEAO/SAO is very common in parts of some countries, with up to 25% of people having the condition in Papua New Guinea, no homozygous individuals for the SEAO/SAO mutation have been described, which led to the supposition that this mutation, and others with similar defective properties, may be lethal.^97^ That this is not always the case was first demonstrated in 1994 in certain Japanese black cattle—bred for their prized Wagyu meat. These animals have a nonsense mutation for band 3 and their red cells totally lack band 3.^98^ Consequently, they cannot carry out anion exchange, compromising CO_2_ carriage, and they are also mechanically unstable.

The Japanese cattle band 3 mutants are moderately anaemic with evidence of increased erythropoiesis though excessive intravascular haemolysis is absent.^98^ Some die as calves, others have retarded growth, but many live until adulthood. CO_2_ transport appears only mildly disrupted, with a modest increase in the partial pressure of CO_2_ required to enable its transport. They show a surprisingly mild respiratory acidosis and only a slightly lowered urine pH. However, the animals are not heavily exercised when restrictions on the ability to transport CO_2_ would be more manifest. The meat from these cattle has a particularly desirable fat marbling. As the intramuscular pH is a key factor in tenderising meat post‐mortem, it is also interesting to speculate on the extent to which their altered red cell phenotype contributes.

Rather than compromising CO_2_ transport, the absence of red cell band 3 mainly impacts upon cytoskeletal integrity. Red cells in affected cattle show a pronounced hereditary spherocytosis and anisocytosis, probably due to disruption of the spectrin network and membrane shedding, with a slightly raised mean corpuscular volume and reduced haemoglobin concentration.^98^ The copy number of proteins associated with band 3—ankyrin, band 4.2, spectrin and actin—is reduced.^98^ Cell integrity seems to be taken over by the glycophorin‐band 4.1‐spectrin associations but loss of membrane results in a shape transition from discocytes to stomatocytes and spherocytes and other abnormal shapes.

A few years after the report in Japanese cattle, a mutant mouse line was created lacking band 3.^99^, ^100^ These, too, were also found to be non‐lethal with some surviving to adulthood, although they appear more severely affected than the cattle. The mice show significant anaemia, spherocytosis and haemolysis. Unlike cattle, their red cells are not deficient in band 4.1 or spectrin, but ankyrin levels are reduced and band 4.2 is absent. Cytoskeletal architecture is near normal with the quasi‐hexagonal arrangement of spectrin still clearly visible^99^, ^101^ though membrane loss is considerable. In these mice, the spectrin–glycophorin linkage to the plasma membrane appears to adequately replace that via band 3. A fuller analysis of why these apparent differences with cattle occur would provide a useful comparative study.

More recently, some human patients have been reported with no band 3, for example, the ‘Coimbra’ and ‘Vienna’ mutants,^102^, ^103^, ^104^ and individuals are able to survive to at least early adulthood. It, therefore, appears that band 3 is not essential for the survival of human red cells nor the presence of some sort of cytoskeleton. Nevertheless, it is critical for normal integrity of the red cell membrane and also for normal red cell erythropoiesis^105^ and other red cell functions.^87^, ^88^, ^92^

## PATHOPHYSIOLOGY

The preceding sections illustrate how comparative studies have contributed to our understanding of how the physiological function of pumps and leaks—the Na^+^/K^+^ pump, PMCA, band 3, the Gárdos channel, PIEZO1, KCC, cytoskeletal proteins and others—contribute towards normal red cell behaviour, volume and shape and longevity. However, what happens when they go wrong?

Compensation for mild dysfunctions may be possible, but ultimately severe abnormalities will result in red cell destruction. In these conditions, unbalanced cation movement across the membrane increases or decreases red cell volume, inducing haemolysis or cell removal. Thus, ‘[w]hile ensuring cell stability under normal conditions,…… altered membrane transport becomes a serious liability’.^53^ Mutations affecting the permeability of membrane proteins may be responsible and underlie some of the rarer haemolytic anaemias, more comprehensive accounts of which can be found elsewhere.^44^, ^58^, ^59^, ^94^, ^106^

**FIGURE 5:**
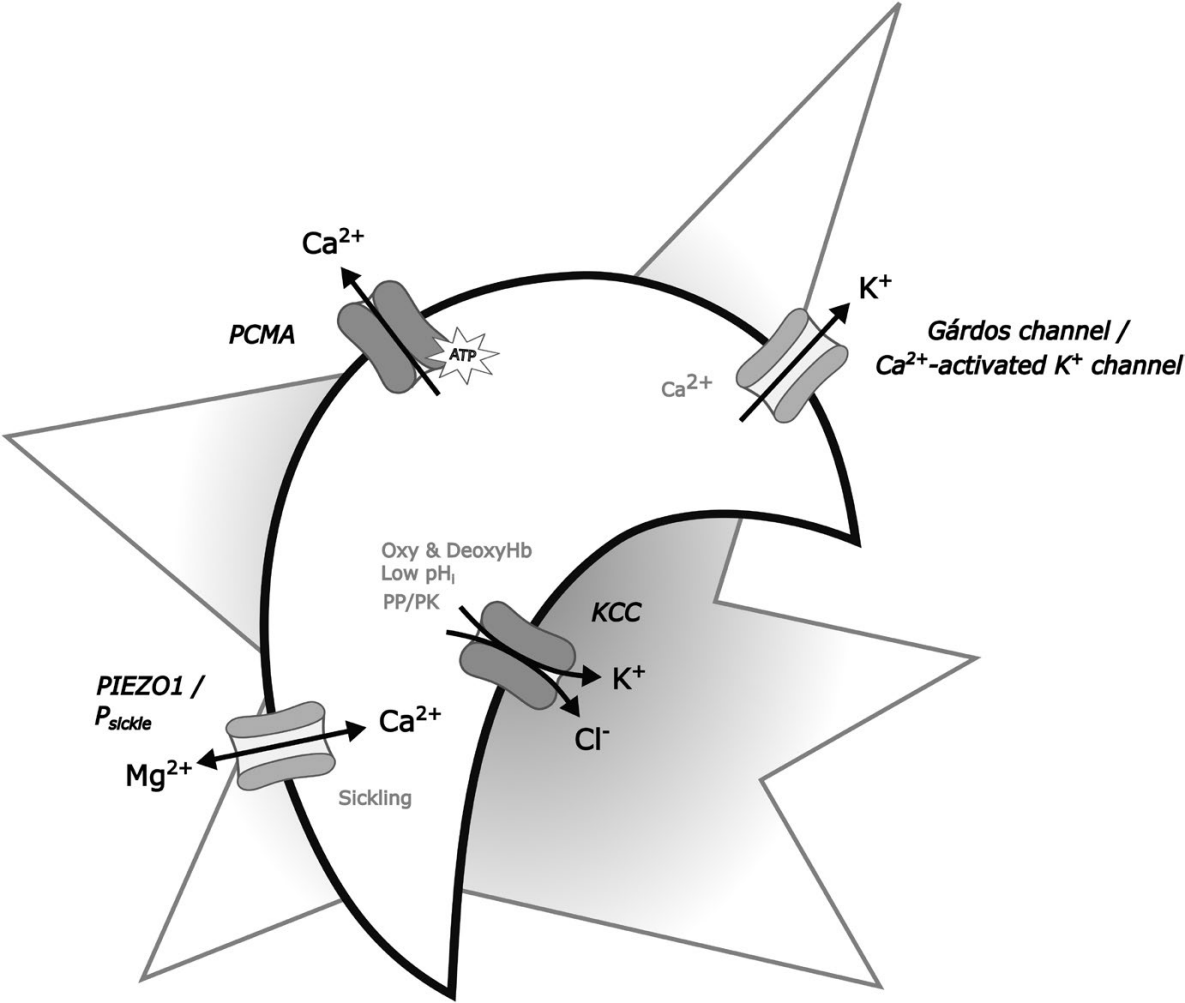
Altered membrane transport in sickle cell disease. Red cells from patients with sickle cell disease (SCD) contain the mutated Hb, HbS, which polymerises on deoxygenation. Red cell shape change follows together with other cellular abnormalities including increased cation permeability.^44^, ^107^, ^109^ Ca^2+^ entry, Mg^2+^ loss, dehydration and lipid scrambling contribute to pathogenesis. In oxygenated sickle cells, the main problem is an increased activity of the KCl cotransporter (KCC).^110^ In deoxygenated sickle cells, KCC remains abnormally active^111^ and several other transporters also manifest. Red cell distortion during sickling increases activity of PIEZO1, and Ca^2+^ entry through this mechanosensitive channel activates the Ca^2+^‐activated K^+^ channel or Gárdos channel,^56^ causing a rapid loss of K^+^ and Cl^−^ through separate ion channels. Ca^2+^ entry also mediates Ca^2+^‐dependent lipid scrambling and exposure of phosphatidylserine (PS).^112^ Mg^2+^ loss through PIEZO1 will further stimulate KCC, while intracellular acidification following any KCl loss increases activity further.^44^ Interestingly, WNK1 activity is implicated in the control of cation‐chloride cotransporters (CCCs)^71^ and disruption of macromolecular crowding through HbS polymerisation, and the inability of HbS polymers to bind to band 3 may contribute to the abnormal high activity and altered O_2_ dependence of KCC in red cells from HbSS SCD patients,^111^ c.f. those from patients with HbSC disease^113^—see text for details.

An example of considerable clinical significance is the various genotypes of sickle cell disease (SCD). In this case, mutations in Hb, rather than in transport proteins, nevertheless have profound effects on red cell permeability. The changes have been much studied.^44^, ^107^, ^108^ In SCD, deoxygenation, HbS polymerisation and sickling probably result in over activity of PIEZO1. The result is Ca^2+^ entry and Mg^2+^ loss which has profound effects on volume homeostasis. Ca^2+^ overload activates the Gárdos channel. Mg^2+^ loss further stimulates an overactive and abnormally regulated KCC activity (as summarised in Figure 5 and Table 2). Other potential effects through altered macromolecular crowding from HbS insolubility and subsequent interference with the WNK pathway remain largely uninvestigated, especially with regard to KCC activity. The interplay between KCC and the other transport systems in SCD raises the possibility of positive feedback,^44^ resulting in rapid red cell dehydration and enhanced HbS polymer formation, contributing to pathogenesis.

This is an area, however, in which there is a paucity of comparative studies. Rare human diseases, nevertheless, attract considerable resources into their understanding—those in animals tend not to. Potential analogues to some of these human diseases exist—for example, stomatocytosis in certain dog breeds,^114^ the consequences of Hb polymerisation and red cell sickling in deer^115^ and also certain fishes^116^ or poikilocytosis in Angora goats^117^—have been little studied. In all probability, our understanding of these pathophysiological conditions would also benefit from more comparative studies.

## CONCLUSION

This review highlights how studies on animal red cells have enriched our understanding of the behaviour of those from humans, contributing to our knowledge of ion and water homeostasis and cytoskeletal integrity. The pump–leak model for volume stability is indebted to work on sheep red cells. Studies in several species including trout have been useful for detailing how the co‐ordinated behaviour of a number of red cell transport proteins is involved in volume homeostasis and other functions including how phosphorylation pathways control the activity of the cation‐chloride cotransporters. Red cells from carnivores were historically important for developing theories on macromolecular crowding. Cattle red cells have helped substantiate that band 3 may not be essential for red cell existence. Work in many other species has informed our understanding of red cell physiology. Nevertheless, animal models of pathophysiology warrant more attention. As Krogh noted nearly a century ago: ‘For a large number of problems there will be some animal of choice…on which it can be most conveniently studied’.^118^

## AUTHOR CONTRIBUTIONS

Kathleen M. Connolly, Pengyi Ding, Rasiqh Wadud, David C. Rees, John N. Brewin and John S. Gibson, all helped write the manuscript. Figures were drawn by Kathleen M. Connolly, with suggestions from John S. Gibson.

## FUNDING INFORMATION

This work is supported by a grant from the Wellcome Trust (G130092).

## CONFLICT OF INTEREST STATEMENT

The authors have no conflicts of interest to disclose.

## Data Availability

The data that support the findings of this study are available on request from the corresponding author. The data are not publicly available due to privacy or ethical restrictions.
